# Evolutionary Time-Scale of the Begomoviruses: Evidence from Integrated Sequences in the *Nicotiana* Genome

**DOI:** 10.1371/journal.pone.0019193

**Published:** 2011-05-16

**Authors:** Pierre Lefeuvre, Gordon W. Harkins, Jean-Michel Lett, Rob W. Briddon, Mark W. Chase, Benoit Moury, Darren P. Martin

**Affiliations:** 1 CIRAD, UMR 53 PVBMT CIRAD-Université de la Réunion, Pôle de Protection des Plantes, Saint Pierre, La Réunion, France; 2 South African National Bioinformatics Institute, University of the Western Cape, Bellville, South Africa; 3 National Institute for Biotechnology and Genetic Engineering, Faisalabad, Pakistan; 4 Jodrell Laboratory, Royal Botanic Gardens, Kew, United Kingdom; 5 INRA Avignon, UR407 Pathologie Végétale, Domaine St Maurice, Montfavet, France; 6 Computational Biology Group, Institute of Infectious Diseases and Molecular Medicine, University of Cape Town, Cape Town, South Africa; American Museum of Natural History, United States of America

## Abstract

Despite having single stranded DNA genomes that are replicated by host DNA polymerases, viruses in the family *Geminiviridae* are apparently evolving as rapidly as some RNA viruses. The observed substitution rates of geminiviruses in the genera *Begomovirus* and *Mastrevirus* are so high that the entire family could conceivably have originated less than a million years ago (MYA). However, the existence of geminivirus related DNA (GRD) integrated within the genomes of various *Nicotiana* species suggests that the geminiviruses probably originated >10 MYA. Some have even suggested that a distinct New-World (NW) lineage of begomoviruses may have arisen following the separation by continental drift of African and American proto-begomoviruses ∼110 MYA. We evaluate these various geminivirus origin hypotheses using Bayesian coalescent-based approaches to date firstly the *Nicotiana* GRD integration events, and then the divergence of the NW and Old-World (OW) begomoviruses. Besides rejecting the possibility of a<2 MYA OW-NW begomovirus split, we could also discount that it may have occurred concomitantly with the breakup of Gondwanaland 110 MYA. Although we could only confidently narrow the date of the split down to between 2 and 80 MYA, the most plausible (and best supported) date for the split is between 20 and 30 MYA – a time when global cooling ended the dispersal of temperate species between Asia and North America via the Beringian land bridge.

## Introduction

Virus populations are generally dynamic ensembles of individual lineages, each with distinctive genetic diversities, geographical distributions and host ranges. Combining information on the phylogenetic relationships of virus lineages, the sampling times of individual viruses, their sampling locations and their host species can both reveal the historical processes that have shaped their evolution and enable estimation of the time-scales over which these processes have operated [1], [2]. Among many other examples, such a “phylodynamic” approach has provided key insights into the emergence of important crop pathogens along with agriculture during the Neolithic period [3], the West-African origin around the start of the 20^th^ century of HIV-1M (the virus lineage driving the global AIDS pandemic) [4], and the origin and global dissemination routes of H1N1 influenza viruses during the recent swine flu pandemic [5].

An essential component of such studies is the accurate inference of virus evolutionary rates as these enable the use of phylogenetic trees to date important evolutionary events such as host switches, geographical range expansions and changes in population sizes. Different approaches can be used to infer the rate at which mutations arise (the mutation rate) and become fixed within a population (the substitution rate) during virus evolution (reviewed in [6], [7]). These include: (i) the direct estimation of mutation and substitution rates using experiments where virus genomes with either known or accurately inferable sequences are used to initiate infections and are then compared with virus sequences subsequently sampled from these infections over time-periods ranging from days to years [8]–[10]; (ii) the direct estimation of substitution rates from natural sequences sampled over extended time periods by correlating degrees of divergence from ancestral sequences with sampling dates [11], [12]; (iii) the indirect estimation of substitution rates by associating the phylogenetic divergence of virus lineages that have different host or geographical ranges with either the known divergence dates of their host species (so-called virus-host co-divergence) or the dates when the geographical ranges of virus lineages diverged (for example, when one of the lineages was either introduced into a new isolated environment such as an island or a different continent) [13]–[15]; and (iv) the indirect estimation of substitution rates based on the relationships of contemporary virus sequences to those of “fossil” virus sequences that have become integrated into host genomes [7], [16]–[19].

Importantly, each of these viral substitution rate inference approaches has its own shortcomings [6], [7]. It is problematic, for example, to use substitution rate estimates from either short-term experiments or natural sequences sampled over a few decades to date evolutionary events that may have happened thousands or millions of years ago [6], [7]. It is similarly unacceptable to use substitution rates estimated using fossil virus integration events, host species divergences or geographical range splits that occurred millions of years ago to estimate the divergence times of virus lineages that only split a few tens or hundreds of years ago. The crux of the problem is that every site within a virus genome probably evolves at a different rate, with some sites evolving very slowly under strong negative selection (selection that disfavours change) and others evolving more rapidly under either weaker negative selection, neutral genetic drift or positive selection (selection favouring change). Also, not only do substitution rates probably vary from site to site across virus genomes, but they can also differ between related virus lineages in ways for which it can be difficult to account for.

Accurately dating viral evolutionary events can therefore be a complex and sometimes controversial task especially when it requires the extrapolation of evolutionary rates either beyond the time-scales over which they are valid or to virus genotypes or species that are only distantly related to those for which the rates were inferred. Such difficulties are exemplified by recent studies aimed at determining the rate at which members of the single stranded DNA virus family, *Geminiviridae*, are evolving. These viruses are arthropod-borne and are known to display both relatively high short-term substitution [10], [11], [20] and recombination rates [21], [22]. Whereas experiments and analyses of virus sequences sampled over a few decades have indicated that at least some viruses in the *Mastrevirus* and *Begomovirus* genera of the *Geminiviridae* have substitution rates that are similar to those of rapidly evolving RNA viruses [7]–[11], [23], [24], they have been unable to definitively discredit alternative claims such as (1) geminiviruses having much slower long-term evolution rates such that they might have co-diverged with either their host or vector species [13], [25] or (2) genetically distinct geminivirus lineages in the Americas having diverged from their nearest relatives in the rest of the world following the breakup of Gondwanaland ∼100 MYA [23].

Interestingly, repetitive begomovirus-like sequences, called geminivirus related DNA (GRD) elements, have been discovered integrated within the genomes of various *Nicotiana* species [26]–[29]. There has recently been a flurry of interest in such “fossilised” virus sequences when it was realised that they could potentially be used to determine the long-term substitution rates of their contemporary relatives [16]–[19], [30]. Accordingly, Gibbs et al. [7], have used time-calibrated *Nicotiana* phylogenetic trees both to infer that begomoviruses were present in the Americas at least 1.9 million years ago (MYA), and to estimate that the long-term substitution rate of the begomovirus CP gene is lower than 6×10^−7^ substitutions per site per year (subs/site/year) – a rate that is between two and four orders of magnitude lower than the short-term nucleotide substitution rates estimated for the CP genes of both mastreviruses [23] and begomoviruses [11]. Such low long-term substitution rates are clearly consistent with the hypothesis that some geminivirus species might be co-diverging with either their vector or host species [13], [25].

Here we attempt to date more precisely the two separate insertions of GRD sequences into the genomes of ancestral *Nicotiana* species. We then use inferred ancestral GRD sequences together with the homologous sequences of contemporary begomoviruses to directly compare short- and long-term begomovirus substitution rate estimates. Finally, we evaluate various geminivirus origin hypotheses by determining the date when the distinct New-World (NW) begomovirus lineage diverged from the various Old-World (OW) begomovirus lineages.

## Materials and Methods

### Virus sequence data

All of the GRD sequences that have been identified within the genomes of *Nicotiana tabacum* (14 sequences), *N. tomentosa* (1 sequence), *N. tomentosiformis* (5 sequences), and *N. kawakamii* (3 sequences) were obtained either from GenBank in June 2009 or newly sequenced (Accession numbers HQ331526 to HQ331528) from fresh material using 454 GS FLX Instrument and Titanium reagents (Roche Diagnostics) at the Natural Environment Research Council (NERC) Biomolecular Analysis Facility (Liverpool, UK). These GRD elements have arisen through at least two and possibly three independent integration events during the evolution of these four *Nicotiana* species [26], [28], [31]. Depending on the integration event from which they were derived, the elements have been classified into three groups called GRD2, GRD3 and GRD5. GRD2 is represented by only a single sequence within *N. tomentosa* and this sequence was therefore not included in any further analyses. All of the GRD3 and GRD5 sequences were aligned along with seven contemporary begomovirus sequences (accession numbers: AJ965339, AY049226, AY083351, EF417915, EU822322, FJ222587 and FJ972767) using the Clustal-W sub-alignment tool [32] available in MEGA4 [33]. Reconstruction of the most recent common ancestor (MRCA) of the GRD3 elements and the MRCA of the GRD5 elements was conducted using MrBayes v3.1.2 [34] and the ancestral sequence reconstruction features available in RDP3 [35]. Only sites that could be inferred with posterior state probabilities of >0.95 were retained for further analysis (resulting in 8 and 5 sites being discounted from the GRD3 and GRD5 sequence alignments respectively).

The two inferred GRD ancestral sequences were aligned using POA [36] to a set of 57 representative OW (n = 46) and NW (n = 11) begomoviruses sequences (Table S1). These 57 viruses were chosen, based on their relative diversity, as being representative of all the begomovirus sequences available in Genbank in July 2008. The apparent bias in favour of OW viruses is simply due to the OW viruses being more diverse than their NW counterparts. This alignment was edited by eye and using the Clustal-W [32] based sub-alignment tool in Mega4 [33]. After removing poorly aligned columns we obtained an alignment containing 848 nucleotides that was used for the estimation of long-term begomovirus substitution rates.

**Figure 1:**
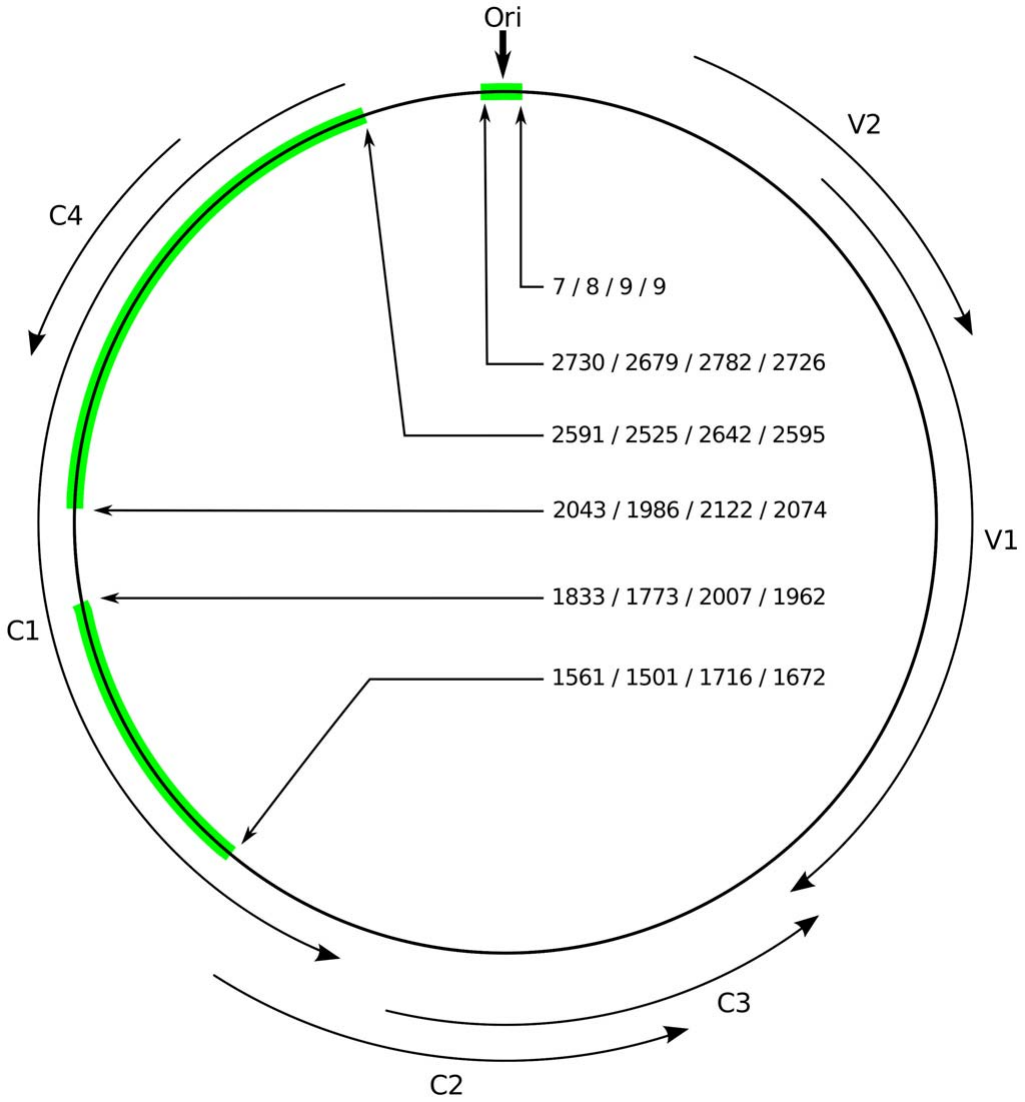
Representation of the coordinates of the GRD homologous sequences (in green) within the EACMV genome (accession number AJ717572). ORFs are represented using curved black arrows. The origin of virion strand replication is marked with a vertical black arrow. Position coordinates of the GRD homologous sequences are given relative to the SSRV (EU244913), MSV (FJ882103), EACMV (AJ717524) and TYLCV (X63015) genomes.

In order to estimate short-term substitution rates we further assembled four different temporally structured (i.e. with sampling dates spanning multiple years) datasets consisting of *Tomato yellow leaf curl virus* (TYLCV, a begomovirus species), *East African cassava mosaic virus* (EACMV, another begomovirus species), *Sugarcane streak Reunion virus* (SSRV, a mastrevirus species), and *Maize streak virus* (MSV, another mastrevirus species) sequences. The TYLCV datasets (decribed in [11]), EACMV datasets (described in [20]), SSRV and MSV datasets (described in [10] and [23]) were obtained from GenBank. These datasets were, however, trimmed to only contain fragments homologous to those found in the GRD sequence dataset (Figure 1). Details on the composition and lengths of these alignments are given in Table 1 and Table S2.

**Table 1:** Short term evolution rate estimations.

dataset	TYLCV	EACMV	SSRV	MSV
number of sequences	56	76	81	124
sequences length	1116	1110	836	828
time span of sequences	1988–2006	1995–2002	1987–2008	1979–2008
Mean substitution rate*	4.04E-4	1.56E-3	1.82E-3	3.87E-4
HPD 95% lower	2.44E-5	1.46E-5	6.41E-4	2.12E-4
HPD 95% upper	1.01E-3	4.94E-3	3.25E-3	5.6E-4

*in substitutions per site per year.

### *Nicotiana* gene sequence data

The *matK*, *ndhF*, *trnL-F* inter genic spacer (IGS), *trnS-G* IGS, and the internal transcribed spacer (ITS) regions of nuclear ribosomal DNA (nrDNA) sequences of various *Nicotiana* species (Table S3, also described in [37] and [38]) were obtained from Genbank, and were aligned using the Clustal-W [32] based alignment tool in Mega4 [33] and by eye to produce a *Nicotiana* dataset.

### Estimation of nucleotide substitution rates

We used BEAST v1.4.8 and v1.5.2 [39] to infer the date of the split between the OW and NW begomoviruses using different approaches to estimate nucleotide substitution rates. In the first approach we estimated nucleotide substitution rates from begomovirus and mastrevirus sequences that have been sampled over a number of years (referred as our “short-term substitution rate” estimates) and used these rates to infer credible bounds on the age of the MRCA of the OW and NW begomoviruses. As the short-term substitution rates were estimated with sequences sampled over comparatively short time periods (30 years at most) relative to the time since the OW and NW begomovirus split (a period potentially spanning millions of years), one could expect these rates to be misleading. This is because the short-term rates are expected to be faster than long-term rates due to their being both upwardly biased by transient substitutions that do not become fixed over the long-term, and blind to the true rates of change at slowly evolving, but apparently invariant, nucleotide sites at which negative selection is acting.

To deal with this issue, in our second approach we attempted to use the integrated GRD sequences (or at least the inferred ancestral sequences thereof) as though they were sequences sampled in the extremely distant past. Specifically, we constructed datasets with extreme temporal structure by treating contemporary begomovirus sequences as having been sampled in the last year and treating the inferred GRD MRCA sequences as having been sampled at the dates when these sequences were inferred to have integrated into ancestral *Nicotiana* genomes.

For every analysis described below, we dated ancestral sequences and estimated nucleotide substitution rates using the Bayesian relaxed-clock approach implemented in BEAST with the following general methodology: After choosing the best nucleotide substitution model using the ModelTest-like [40] approach implemented in RDP3 [35], substitution rates and MRCA estimates were made with BEAST using three molecular clock models (strict-clock, uncorrelated exponential relaxed-clock and uncorrelated lognormal relaxed-clock) and various demographic models (constant, expansion, exponential and Bayesian skyline models for the virus population datasets or Yule and birth-death models for the *Nicotiana* species datasets). Wherever possible all runs were continued until convergence of the various model parameters as adjudged using Tracer v.1.5 (available at http://tree.bio.ed.ac.uk/software/tracer) by manual inspection of parameter estimate traces and the achievement of suitable effective sample sizes for these parameter estimates. We identified the best fit clock and demographic models by (1) using a Bayes Factor (BF) test [41], [42] of the marginal tree likelihoods using Tracer v1.5 and (2) by manual inspection of the estimated standard deviation of the uncorrelated log-normal clock parameter (ucld.stdev, a measure of the degree to which nucleotide substitution rates vary between branches, which provides an indication of whether the strict-clock should be accepted or rejected).

Having identified the best-fit clock and demographic models (see Table S4), we carried out a suitable number of independent MCMC runs to achieve convergence both with effective sample size estimates that usually exceeded 200 and with stable distributions for key model parameters (visualised with Tracer v1.5). Summary statistics for tree nodes representing key ancestral sequences (for example, the MRCA of the OW and NW begomoviruses or the MRCA of the *Nicotiana* species carrying the different GRD elements) were obtained using Tracer v1.5. Where appropriate, maximum clade credibility (MCC) trees were constructed using TreeAnnotator [39] and visualized using FigTree v.1.3 (available at http://tree.bio.ed.ac.uk/software/figtree).

### Dating the MRCA of the OW and NW begomoviruses

We estimated the time since the MRCA (t_MRCA_) of the OW and NW begomoviruses using substitution rates determined using four independent geminivirus datasets (including two begomovirus datasets - EACMV and TYLCV – and two mastrevirus datasets – SSRV and MSV; Table 1). These substitution rates were subsequently employed as evolutionary rate priors when determining the approximate age of the OW-NW begomovirus split using BEAST.

**Figure 2:**
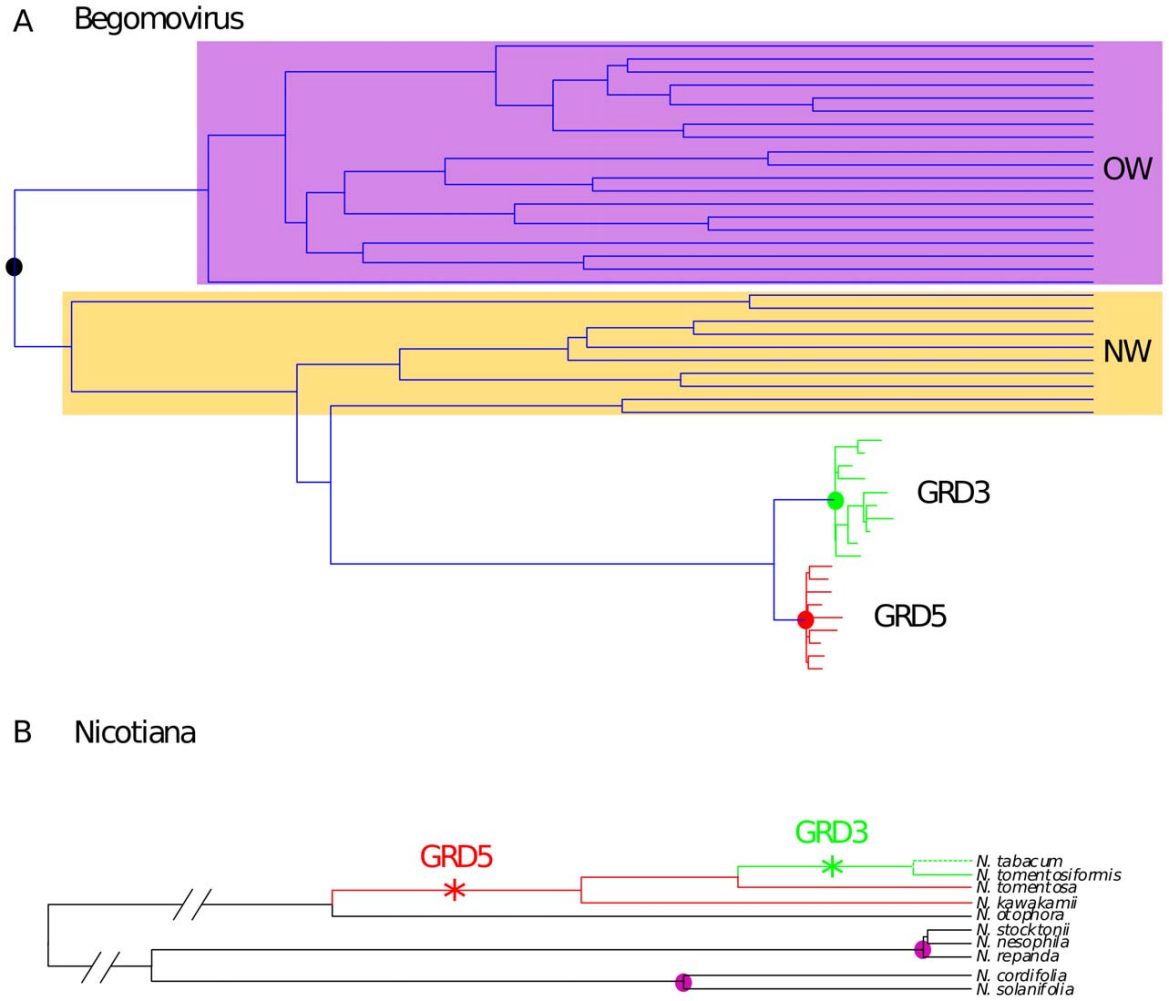
Schematic representation of dataset phylogenies and the calibration points used to estimate begomovirus long-term nucleotide substitution rates. (A) Begomovirus phylogentic tree with the Old-World (OW) and New-World (NW) sequences indicated in purple and orange respectively. The node representing the most recent common ancestor of the OW and NW begomoviruses is indicated by a black circle. GRD3 (green) and GRD5 (red) sequences are grouped with the New World begomoviruses with the nodes representing ancestral integrons indicated with green and red circles. (B) Partial representation of the *Nicotiana* phylogenetic tree that was used to infer the dates of GRD sequence integration events. The branches labelled with stars represent ancestral lineages within which GRD5 and GRD3 integrations occurred. Circles in purple are nodes that can be dated based on *Nicoticana* speciation events associated with the geographical isolation of certain *Nicotiana* lineages on Pacific islands with inferable geological ages.

In order to use the integrated begomovirus-like GRD sequences to estimate the long-term begomovirus substitution rate it was necessary to first determine the date when the GRD3 and GRD5 sequences became integrated into the *Nicotiana* genome. It is important to first point out here that our analysis relied on the assumption of a single integration event for each of the GRD3 and GRD5 sequences within an ancestral *Nicotiana* genome. If these GRD sequences had been transferred multiple times between different *Nicotiana* species by hybridization (a remote but real possibility), our estimates of their integration times could be misleading. We therefore used two approaches to estimate this date: one that would yield a completely incorrect date if the GRD sequences had been dispersed amongst the *Nicotiana* species by hybridization, and another that was less dependent on the occurrence of a single integration event. In the first method (which was potentially confounded by lateral GRD transfers), we used BEAST to estimate the age of the nodes within the *Nicotiana* phylogenetic tree that bounded the tree branches where the GRD integration events were believed to have occurred (Figure 2; [43]). This was achievable because two other nodes within the *Nicotiana* tree have been previously dated based on speciation events attributable to the geographical isolation of *Nicotiana* populations on Pacific Ocean islands with known geological ages [43]. These BEAST analyses were carried out using data partitions that permitted independent substitution rates for the *ndhF*+*matK* sequences (coding plastid sequences for which an independent 1+2 and 3 codon position model was used), *trnL-F* IGS+*trnS-G* IGS sequences(non-coding plastidsequences for which a standard nucleotide substitution model was used) and ITS nrDNA sequences (non-coding nuclear sequences for which a standard nucleotide substitution model was used). This approach provided an estimate of the time interval over which the GRD3 and GRD5 integrations most plausibly occurred.

In a second, potentially more accurate approach that was also less confoundable by GRD lateral transfers, we estimated the GRD integration times using the neutral substitution rate inferred for *Nicotiana* ITS nrDNA sequences. Our assumption that, following integration into the *Nicotiana* genome, the dominant force driving the observable diversity within the GRD sequences has been neutral genetic drift is supported by selection analyses of the GRD sequences which have strongly suggested (albeit with only a small number of sequences), that following integration, the integrons have been subject to nearly identical synonymous and non-synonymous substitution rates [29]. Rather than simply dating the internal nodes in the *Nicotiana* tree that bounded the integration event, this second approach dated the integration events as being the date of the most recent common ancestors of the GRD3 and GRD5 groups. To this end, we first estimated the ITS nrDNA substitution rate in BEAST (as described above) using the inferred dates when the two *Nicotiana* species became isolated on different Pacific Ocean islands. We then used this inferred nucleotide substitution rate as a prior to date the MRCAs of the GRD sequences using BEAST.

With the GRD integration dates in hand we attempted to determine the t_MRCA_ of the OW and NW begomoviruses using BEAST. Since the *Nicotiana* genus was until recently restricted to the NW, a prior assumption in these analyses (which is supported by independent phylogenetic analyses [26]) was that the GRD and NW begomoviruses share a more recent common ancestor than either of these does with the OW begomoviruses. Whereas the contemporary OW and NW begomovirus sequences used in these analyses were given sampling dates of 0 time units ago, those of the GRD sequences were given sampling times corresponding to the median, 95% upper and 95% lower highest probability density (HPD) intervals of the GRD3 and GRD5 integration dates.

## Results and Discussion

### Dating the OW and NW begomovirus MRCA using short-term nucleotide substitution rate estimates

We first analysed four geminivirus datasets (TYLCV, EACMV, MSV, and SSRV) that have been used in the past to infer short-term geminivirus nucleotide substitution rates. We focused specifically on the genome regions corresponding to those occurring in the GRD elements found integrated within the genomes of various *Nicotiana* species. In all four analyses, BF tests indicated that the relaxed-clock model fitted the data better than a strict-clock model (see Table S4). Although BF tests indicated that the uncorrelated exponential relaxed-clock fit the MSV data best (in comparison to the uncorrelated log-normal relaxed-clock), there were no significant differences between the demographic models tested (constant population size, expansion growth, exponential growth and Bayesian skyline plot). Mean substitution rates for the portion of these datasets that corresponded with the GRD elements ranged from 1.8×10^−3^ subs/site/year for the mastrevirus, SSRV, to 3.9×10^−4^ subs/site/year for the mastrevirus, MSV. Whereas the 95% HPD estimates of the MSV and SSRV isolates did not overlap, the MSV, TYLCV and EACMV intervals did broadly overlap as did the TYLCV, EACMV and SSRV intervals. It is important to stress here that the SSRV estimate was obtained from a 20-year experiment and is expected to be slightly higher than the MSV, TYLCV and EACMV estimates, which were determined from sequences sampled from nature. This is because it is expected that the small experimental population represented in the analysed SSRV dataset would have been subject to less effective purifying selection than the larger natural populations represented in the other three datasets [6].

Despite small differences between the substitution rates estimated with these various geminivirus species, they are all nevertheless fairly congruent with those published previously for these species [10], [11], [20]. This implies that across the entire geminivirus family the GRD-like portion of the geminivirus genome displays relatively high substitution rates that are both approximately equivalent to those estimated in other parts of geminivirus genomes and within an order of magnitude of those estimated for RNA viruses [6], [44].

When it came to dating the t_MRCA_ of the OW and NW begomoviruses we focused primarily on the substitution rate estimated with the TYLCV dataset (4.04×10^−4^ subs/site/year with 95% HPD ranging from 2.44×10^−5^ to 1.08×10^−3^). There were two reasons that we thought this was the best of the four estimates that we made. The first was that the two mastrevirus datasets (MSV and SSRV) are almost certainly less informative than the two begomovirus datasets (TYLCV and EACMV) when it comes to dating the MRCA of the OW and NW begomovirus sequences. The second was that the sampling dates of the analysed EACMV sequences spanned only seven years whereas those in the TYLCV dataset spanned 19 years, such that the substitution rates determined with the TYLCV dataset have likely been less affected by transient substitutions that do not eventually become fixed in the population [6]. We hereafter refer to the TYLCV substitution rate estimate as the begomovirus “short-term substitution” (STS) rate.

By applying the estimated STS rate as a prior model specification during reconstruction of a more expansive begomovirus phylogeny in BEAST, we were able to infer the t_MRCA_ of the OW and NW begomoviruses. A set of 57 begomovirus homologues of the GRD sequences (46 OW and 11 NW; Table S1), were used in the analysis. In this analysis the relaxed-clock provided a better fit to the data than the strict-clock (see Table S4) as evidenced by the values obtained for the standard deviation of the uncorrelated relaxed-clock that returned a mean of 0.46 across the independent runs (ranging from 0.36 to 0.60) indicating a relatively high degree of rate variation among lineages. Among the demographic models, none was especially favoured over the others except that the Bayesian skyline plot model was unequivocally rejected. Under those conditions, the mean t_MRCA_ of OW and NW begomoviruses was estimated to be ∼2 370 years with 95% HPD intervals ranging from 405 to 20 686 YA.

It is important to note here that the begomovirus GRD homologues that we have focused our analyses on are within one of the most recombinogenic regions of the begomovirus genome [45]. The probable influence of recombination on our GRD-homologous region substitution rate estimate [46], [47] along with the shorter size of our dataset may explain why substitution rates estimated by Duffy and Holmes [11] using tomato-infecting begomovirus full-genome sequences encompass far lower rate estimates than those determined here (ranging 1.34×10^−6^ to 6.06×10^−4^). Due to the strong possibility that recombination confounded our analysis to a greater degree than that of Duffy and Holmes [11], we suggest that their STS rate estimate is more accurate than ours and, therefore, that the date of the OW-NW begomovirus split inferred from their STS rates, between 722–376 670 YA (95% HPD), is probably also more reliable than ours.

### Dating the GRD integration events

In order to obtain a long-term begomovirus substitution rate estimate for use in dating the MRCA of the OW and NW begomoviruses it was necessary to first date the integration of GRD sequences into the ancestral genomes of various *Nicotiana* species. We achieved this with two approaches. In both approaches we made use of the fact that two nodes of the *Nicotiana* tree have been previously dated based on the genetic differences between *Nicotiana* species found on mainland South America and those found on isolated Pacific islands with known geological ages ([43] and Figure 2b). Whereas in the first approach we dated the nodes of the phylogenetic tree on either side of the branches where the integration events are believed to have occurred (indicated by stars in Figure 2b), in the second approach we estimated the neutral *Nicotiana* substitution rate and used this to infer the time required under neutral genetic drift to produce the degrees of genetic diversity observed in the GRD5 and GRD3 sequences.

Considering that GRD5 sequences have only been found integrated within the genomes of *N. kawakamii*, *N. tomentosiformis* and *N. tabacum* and not in those of any other *Nicotiana* species [28], the initial GRD5 integration most likely occurred in the ancestral *Nicotiana* lineage represented by the branch separating these three *Nicotiana* species from the remainder of the *Nicotiana* phylogeny (indicated by the red star on Figure 2b). GRD3 is apparently only present in the *N. tomentosiformis* and *N. tabacum* genomes, and the integration event therefore most likely occurred on the branch separating those two species from the other *Nicotiana* species (indicated by the green star in Figure 2b). The ages of the sequences at the nodes bounding the branches marking the GRD5 and GRD3 integrations were estimated using BEAST. Whereas the exponential relaxed-clock model fitted the *Nicotiana* data best, there was no significant difference between the Yule and birth-death speciation models (see Table S4 for BF test details). These analyses indicated that the integration events most probably occurred between 1.24 MYA and 4.85 MYA for GRD5 (95% HPD ranging from 7000 YA to 11.22 MYA) and between 0.29 MYA and 0.24 MYA for GRD3 (95% HPD ranging from 9 YA to 1.05 MYA, see the annotated tree in Figure S1).

**Figure 3:**
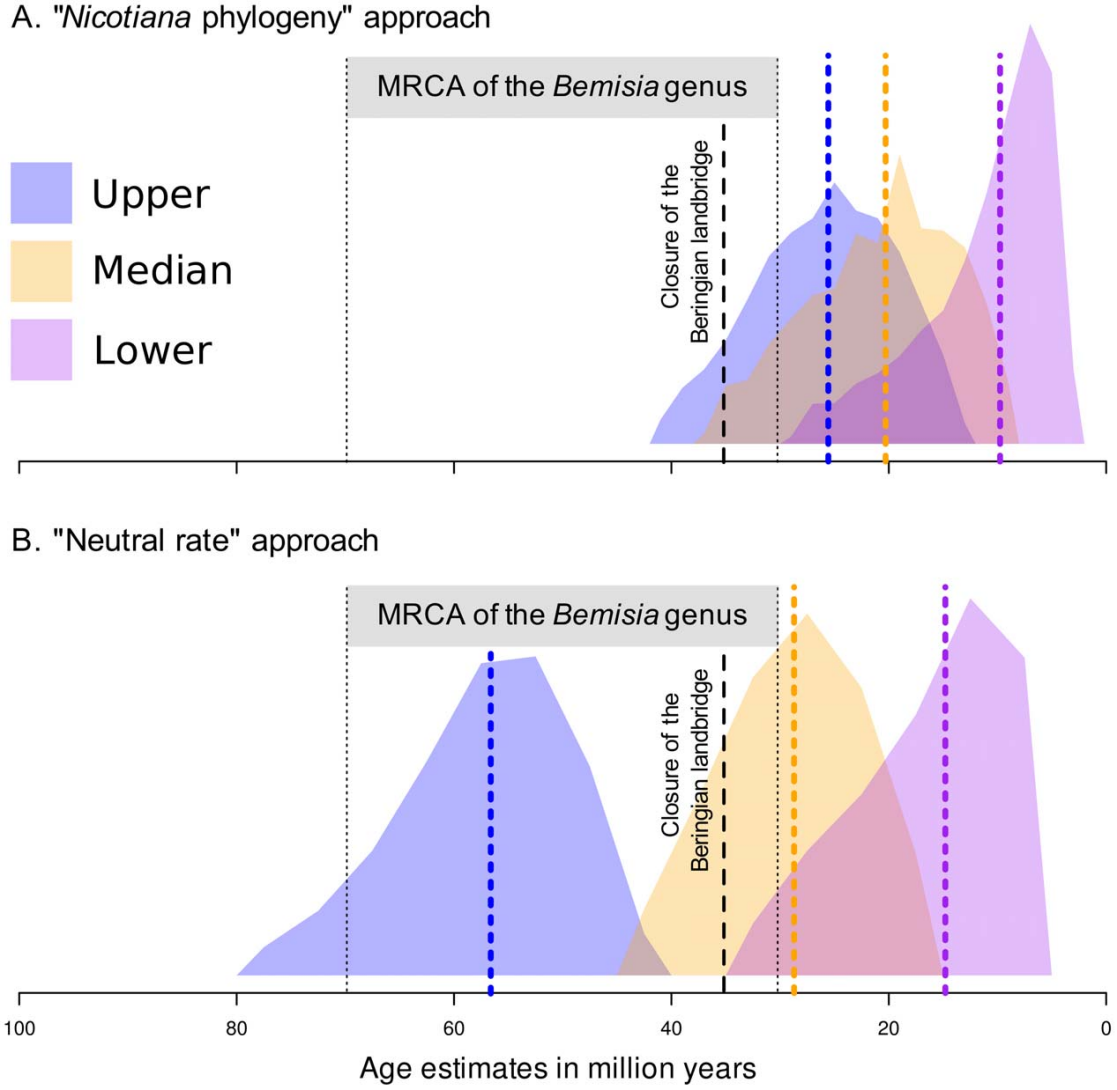
Representation of the posterior probability densities obtained when dating the most recent common ancestor of the Old World and New World begomoviruses using GRD integration times inferred from (A) the dated *Nicotiana* phylogeny (“*Nicotiana* phylogeny approach”) or (B) the estimated time till the most recent common ancestor of the integrated GRD sequences using the neutral substitution rate of *Nicotiana* ITS nrDNA sequences (“Neutral rate approach”). Probability densities obtained with the median, upper 95% HPD limit and lower 95% HPD limit of estimated integration times using two different methods are represented by blue, yellow and purple colours respectively. Vertical dotted lines indicate the median estimated date of each estimate. The grey area represents the time interval during which the genus *Bemisia* originated. The vertical dashed line indicates the point during the Earth's history when global cooling closed the then expansive Beringian land bridge as a pathway permitting the dispersal of tropical plant and animal species between Asia and North America.

In our second approach to dating the GRD integration events we assumed that, following their integration, the GRD sequences have evolved under neutral genetic drift at close to the neutral *Nicotiana* substitution rate [29]. We estimated the date of integration using the substitution rate of the neutrally evolving *Nicotiana* ITS nrDNA sequences (29 sequences, 517 sites). As described above, we used the same geological based dating of two nodes of the *Nicotiana* tree to calibrate the various combinations of clock and speciation models that we tested. The exponential relaxed-clock model fitted the ITS nrDNA data best whereas the Yule and birth-death models fitted the data equally well. The mean neutral *Nicotiana* substitution rate was estimated to be 4.23×10^−9^ subs/site/year with 95% HPD intervals of 1.08×10^−9^ to 8.14×10^−9^ subs/site/year. This rate and its associated 95% HPD interval are well within the bounds of neutral substitution rates estimated for various other angiosperm species (the rates estimated for 29 different species have a mean of 3.41×10^−9^ subs/site/year and range between 3.8×10^−10^ and 1.9×10^−8^ subs/site/year [48]). The inferred *Nicotiana* neutral substitution rate distribution was then used as a substitution rate prior by BEAST to infer the t_MRCA_ of the 10 sampled GRD3 sequences and the 12 sampled GRD5 sequences. After selection of the best-fit clock and demographic models (all the clock models fitted the data equally well, but the exponential population growth model was the least well supported among the demographic models compared; Table S4), the t_MRCA_ of the GRD3 elements was estimated to be 6.1 MYA (median with a 95% HPD between 2 to 24 MYA), and the t_MRCA_ of the GRD5 elements was estimated to be 7.2 MYA (median with 95% HPD between 2.4 to 27 MYA; Figure 3).

Whereas both approaches that we applied yielded estimated dates of the GRD5 integration event that are broadly in agreement with one another, the two estimated dates of the GRD3 integration events do not agree (i.e. we obtained a lower 95% HPD limit of 2 MYA using the neutral *Nicotiana* substitution rate to estimate the t_MRCA_ of GRD3 and a higher 95% HPD limit of 1.05 MYA years when dating the relevant nodes of the *Nicotiana* tree).

### Using the GRD sequences to estimate the long-term begomovirus substitution rate

The inferred ancestral GRD sequences were analysed with BEAST along with 57 contemporary begomovirus sequences. Whereas the ancestral GRD3 and GRD5 sequences were respectively assigned sampling times of the median, upper and lower estimates of the GRD integration (inferred with the “neutral rate” and the “*Nicotiana* phylogeny” approaches), the contemporary sequences were assigned sampling times of 0 MYA.

Of all the tested clock and demographic model combinations, the only BEAST runs that converged were those with a combination of the uncorrelated log-normal relaxed-clock model and the constant population size demographic model. From these runs the long-term begomovirus substitution rate was estimated to be 3.11×10^−8^ subs/site/year (ranging from 1.24×10^−7^ to 9.16×10^−9^ subs/site/year)). Given that the model yielding these estimated rates was the only one that reached convergence, this estimate is probably the best that is achievable with the current data. This rate is substantially lower than the most credible estimates of short-term geminivirus substitution rates (between 1.08×10^−3^ and 2.44×10^−5^ subs/site/year) that we and others have obtained using sequences sampled from natural and experimental virus populations over periods ranging from a few years to three decades. They are in fact far more consistent with those estimated for wheat-, oat- and barley-infecting geminiviruses by Wu et al. [13], based on the possibility that these species may have been co-diverging with their hosts.

### Dating the OW-NW begomovirus split using our long-term substitution rate estimate

The median date of the OW-NW begomovirus split inferred using our estimates of the GRD integration times was 29.6 MYA with the median ITS nrDNA date (95% HPD's between 16.5 MYA and 44.1 MYA) and 20.3 MYA with the median *Nicotiana* date (95% HPD's between 8.3 MYA and 36.1 MYA). Considering the upper and lower 95% HPD bounds of the integration date estimates, the plausible interval within which the OW-NW begomovirus split may have occurred was between 4.6 and 78.2 MYA using the “neutral rate” approach estimated dates and between 2.9 MYA and 40.3 MYA using the “*Nicotiana* phylogeny” estimated dates (Figure 3).

Our estimate that the OW-NW begomovirus split most probably occurred around 20–30 MYA is entirely plausible in that it corresponds with the end of a period in Earth's history between 65 and 35 MYA when there was both a land bridge between Asia and North America (the Beringian land bridge) and a warm global climate such that there existed a continuous band of subtropical vegetation between these continents (reviewed in [49]). This subtropical Beringian land bridge could have conceivably allowed the movement of early begomoviruses between Asia and North America up until ∼35MYA.

It must, however, be emphasised that these most probable estimates of the dates when the OW-NW begomovirus split occurred (i.e. 29.6 and 20.3 MYA) were derived considering only the most probable integration dates of the two GRD elements (i.e. the median estimates of these integration times). Both estimated GRD integration dates had sizable 95% HPD intervals such that had we chosen the higher or lower interval bounds of these integration estimates to determine the date of the OW-NW begomovirus split, the 95% HPD ranges of the split would have been between 2.91 and 78.2 MYA.

Nevertheless, three additional independent lines of evidence suggest that the event that precipitated the divergence of the OW and NW begomoviruses is more likely to have been the closure of the subtropical Beringian land bridge (∼35 MYA) than the fragmentation of Gondwanaland (∼110 MYA). Firstly, the most diverse (and therefore presumably the oldest) group of geminiviruses, the mastreviruses, do not naturally occur in the Americas – a fact indicating that the geminiviruses probably only evolved from their pre-geminivirus progenitor after continental drift separated Africa from South America [50]. Secondly, it is implausible that the OW and NW begomovirus lineages split prior to the speciation of their common vector, *Bemisia tabaci*. The best current estimates for the origin of *B. tabaci* are between 30 and 70 MYA [51]–[53], strongly suggesting that the MRCA of the begomoviruses probably existed more recently than 70 MYA. Thirdly, of all the contemporary OW begomoviruses, those that most closely resemble the NW begomoviruses belong to a divergent lineage that has only ever been found in Southeast Asia [54], supporting the hypothesis that the NW begomoviruses have an Asiatic origin.

Although entirely consistent with the available data, we must again stress that our 20.3 and 29.6 MYA estimates of the OW-NW begomovirus split has a large margin of error. Given that almost all of our various attempts to date the split failed to converge on any meaningful solution, it seems likely that integrated GRD sequences have not retained enough molecular clock information to enable a more precise estimate of the OW-NW begomovirus split. One primary reason that the information encoded in the GRD sequences may not retain a clear temporal signal is that these sequences correspond to what is without a doubt one of the most recombinagenic regions within the genome of begomoviruses [21], [22], [45]. Recombination is known to have a strong influence on molecular clock analyses [46], [47] and can make sequences appear as though they are evolving either more rapidly or slowly than they are in reality. Also, as might be expected of extremely old sequences, the GRD integrons are not particularly closely related to any contemporary begomoviruses. Although they are most similar to and consequently tend to group with NW begomovirus sequences with high statistical support when they are represented in phylogenetic trees, there is always a long branch separating the GRD and begomovirus clades – a factor which, along with the highly recombinagenic nature of begomovirus *rep* sequences, raises the possibility that they are erroneously clustered with the NW begomoviruses. It is plausible that the GRD sequences may have been derived from a now extinct begomovirus lineage that was not more closely related to the present-day NW begomoviruses than they were to present-day OW begomoviruses.

It is also interesting to note here that evidence is emerging that, relative to the geminiviruses, many other agriculturally relevant virus families are extremely young. For example molecular clock analyses of plant virus genomes sampled over the last century have indicated that whereas the genera *Sobemovirus*, *Luteovirus* and *Potyvirus* are probably less than 10 000 years old [1], [3], [55], the genus *Tobamovirus* is probably no more than 100 000 years old [2]. It is possible that, as is apparently the case with divergence times estimated using similar methodology in our analyses, the long-term substitution rates of these other families may have been over-estimated such that their origins might in fact be in the deeper past. In this regard it will be interesting to see whether datable integrons related to these families are uncovered by plant genome sequencing projects.

Similarly, it remains for future plant and begomoviruses genome sequencing projects in the Americas and in Asia (where the presence of NW-like begomoviruses has been observed in the OW [54]) to discover either additional integrated begomovirus sequences or close contemporary viral relatives of GRDs. Such new data might well provide the additional statistical power needed to more accurately resolve the estimated date of the OW-NW begomovirus split. If increased statistical power is achievable with an expanded dataset it might even be possible to ultimately date the MRCA of all the geminiviruses.

### Concluding remarks

Despite our inability to confidently date the MRCA of the NW and OW begomoviruses, some useful information on the origins of the begomoviruses and their evolution can be drawn from our study. One important finding is that the age of the OW-NW begomovirus MRCA cannot be accurately inferred based on short-term nucleotide substitution rates estimated from sequences sampled only in the last 30 years. As is indicated by both our and other attempts to date the GRD integration events, it is extremely probable that begomoviruses were already in the NW at least 2 MYA [7] – an age that is ∼10 times older than the oldest 95% HPD estimates of the OW-NW begomovirus split inferred using our and other short-term substitution rate analyses [11]. As has been recently pointed out by others [6], [13], our results indicate that short-term substitution rate estimates can be misleading when they are extrapolated into the distant past for purposes of dating ancient evolutionary events. Several factors can contribute to such differences between long- and short-term substitution rates including (1) widely varying past natural selection pressures (either positive selection for evolutionary change or negative selection for evolutionary stasis; [7]), (2) occasional erratic increases or decreases in genetic diversity attributable to intermittent population bottlenecks or variations in the rates at which different lineages either die or are born [56] and (3) widely varying substitution rates among nucleotide sites such that rapidly evolving sites inflate short-term evolutionary rate estimates [13]. In summary, one cannot expect that short-term rates of nucleotide substitution inferred from sequences sampled over 30 years from an individual host species can fully recapture the complex long-term evolutionary dynamics of geographically sub-divided viral populations that infect multiple host species.

What then does our analysis tell us about the divergence date of the OW and NW begomoviruses? If one accepts both that the GRD elements following their integration into the *Nicotiana* genome have evolved under neutral genetic drift [29], and that they are remnants of a genuine NW begomovirus lineage (i.e. that they are not derived from (1) a completely independent ancient begomovirus lineage that is no more closely related to contemporary NW begomoviruses than they are to contemporary OW begomoviruses or (2) a related but more divergent initiator protein encoding a DNA fragment from, for example, a phytoplasma plasmid [57], [58]), then our analysis indicates that the NW begomovirus lineage has probably existed in the NW for at least 7.2 million years (the most probable date of the GRD5 integration event). Although our analysis was not powerful enough to exclude the possibility of a 78 MYA OW-NW begomovirus split, we believe that our ∼20.3 and 29 MYA estimates of this date is the most plausible yet proposed. It indicates that the OW-NW begomovirus split may have been directly attributable to a dramatic global cooling event 35 MYA which effectively closed the Bering land bridge between Asia and North America as a dispersal route for tropical plant and animal species (reviewed in [49]). To definitively test this hypothesis using analyses such as we have attempted here, it will most likely require the discovery and analysis of additional fossil geminivirus sequences located within the genomes of other plant species.

## Supporting Information

Figure S1 Maximum clade credibility tree inferred from the *Nicotiana* plastid *matK*, *ndhF*, *trnL-F*, *trnS-G*, and ITS nrDNA sequences. The horizontal scale bar indicates time in millions of years. Error bars are given for nodes of interest. Whereas red bars indicate the two nodes used to time-calibrate the tree, blue bars indicate the nodes used to date the GRD integration events. The numbers associated with nodes indicate the mean ages of GRD integration events as inferred using the Yule and birth-death demographic models, respectively (see the Materials and Methods section for details). Note that in this tree the *N. tabacum* sequence does not group with the other *Nicotiana* species with integrated GRD sequences because *N. tabacum* is a hybrid of *N. tomentosiformis* and *N. sylvestris* and the sequences used to construct this tree were all inherited from its *N. sylvestris* parent.
(TIF)

Table S1 *Begomovirus* accession numbers and descriptions.
(XLS)

Table S2 Short term evolution datasets details.
(XLS)

Table S3 Accession numbers of the sequences used in the *Nicotiana* phylogeny reconstruction.
(XLS)

Table S4 BEAST analyses summary.
(XLS)
