# Supplementary figures and images for: Evolutionary Time-Scale of the Begomoviruses: Evidence from Integrated Sequences in the Nicotiana Genome
Source: PLoS One. 2011 May 16;6(5):e19193. doi: 10.1371/journal.pone.0019193 (PMC3095596; doi:10.1371/journal.pone.0019193)

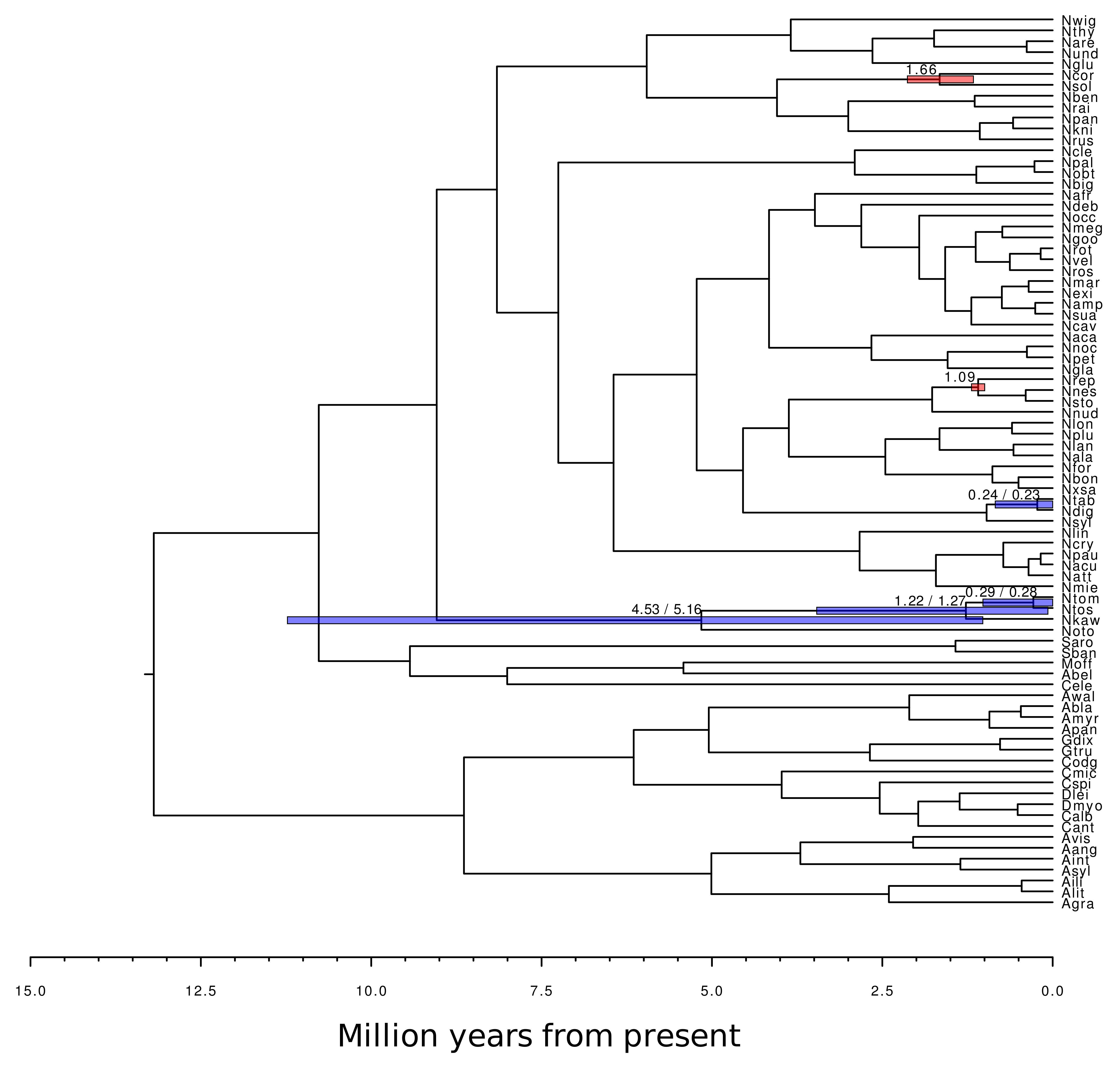

Supplement: Figure S1 — Maximum clade credibility tree inferred from the Nicotiana plastid matK, ndhF, trnL-F, trnS-G, and ITS nrDNA sequences. The horizontal scale bar indicates time in millions of years. Error bars are given for nodes of interest. Whereas red bars indicate the two nodes used to time-calibrate the tree, blue bars indicate the nodes used to date the GRD integration events. The numbers associated with nodes indicate the mean ages of GRD integration events as inferred using the Yule and birth-death demographic models, respectively (see the Materials and Methods section for details). Note that in this tree the N. tabacum sequence does not group with the other Nicotiana species with integrated GRD sequences because N. tabacum is a hybrid of N. tomentosiformis and N. sylvestris and the sequences used to construct this tree were all inherited from its N. sylvestris parent. (TIF) [file pone.0019193.s001.tif]
